# Comparison of Enterocystoplasty and Ureterocystoplasty before Kidney Transplantation

**Published:** 2010-11-01

**Authors:** R. Mahdavi Zafarghandi, A. Zeraati, M. Tavakoli, F. Kalani Moghaddam, M. Mahdavi Zafarghandi

**Affiliations:** *Department of Urology and Renal Transplantation, Imam Reza Hospital, Mashhad University of Medical Sciences, Mashhad, Iran*

**Keywords:** Enterocystoplasty, Ureterocystoplasty, Kidney transplantation, Urinary tract infection

## Abstract

Background: Augmentation cystoplasty before or after renal transplantation is an option for patients with end-stage renal disease who are candidates for renal transplantation and have low capacity and poorly compliant bladders.

Objective: To compare two surgical methods of bladder augmentation—enterocystoplasty (EC) and ureterocystoplasty (UC)—before kidney transplantation, and their outcome with that in kidney recipients who had normal bladder function.

Methods: During a 20-year period (1988–2008), 1406 renal transplantation were performed in our center by our team. In 16 patients having a mean age of 18.8 years, EC (group A) and in 8 with mean age of 11.5 years, UC (group B) were performed before renal transplantation. These two groups were compared with a control group of 30 recipients with normal bladder (group C) with mean age of 15.6 years, for kidney function, graft and patient survival, and the frequency of urinary tract infection (UTI).

Results: There was normal graft function in 11 of group A, 7 of group B, and 24 of group C patients, during a mean follow-up of 73.1 months. The mean±SD serum creatinine in follow-up was 1.72±0.31, 1.37±0.13 and 1.33±0.59 mg/dL in groups A, B and C, respectively. No statistically significant differences were observed among the 3 studied groups in terms of 1-, 5- and 10-year graft and patient survivals. Number of episodes of febrile UTI requiring hospitalization was 23, 6 and 2 in groups A, B and C, respectively. UTI and urosepsis were significantly more frequent in group A than groups B (p=0.025) and C (p=0.001); no significant difference was observed in the frequency between groups B and C (p=0.310).

Conclusion: Both EC and UC are equally recommended before renal transplantation for reconstruction of the lower urinary tract; use of each method should be individualized depending on specific conditions of recipient.

## INTRODUCTION

Structural urologic abnormalities causing dysfunction of the lower urinary tract which lead to end-stage renal disease (ESRD) constitute 15% of patients in adult population and up to 20%–30% in pediatric patients [1]. In patients with ESRD who are candidates for renal transplantation and have low capacity and poorly compliant bladders and who does not respond to clinical measures, augmentation cystoplasty should be performed to decrease intravesical pressure and preserve continence [2].

For this purpose, augmentation cystoplasty using either a segment of intestine (enterocystoplasty (EC)) or dilated ureter (ureterocystoplasty (UC)), before or after renal transplantation, is a therapeutic modality [3-5].

We conducted this study to compare the outcome of EC and UC before kidney transplantation, and their outcomes with that in kidney recipients who had normal bladder function.

We compared the outcome of the two methods of augmentation cystoplasty—enterocystoplasty and ureterocystoplasty—and compared the outcome of these two methods with that of transplant patients with normal bladder function.

## PATIENTS AND METHODS

During a 20-year period (1988–2008), 1406 renal transplantations were performed by our team in Imam Reza Hospital, Mashhad University of Medical Sciences, Mashhad, northeastern Iran. Out of these patients, 24 had undergone augmentation cystoplasty before renal transplantation due to dysfunction of the lower urinary tract. Of these 24 patients, for 16 (11 male and 5 female) underwent EC (group A) using ileum (n=16), colon (n=2) or sigmoid (n=1). The mean age of these patients at time of transplantation, was 18.8 (range: 7–32) years. The etiologies of bladder dysfunction in this group included neurogenic bladder with severe hyper-reflexia (n=12), contracted bladder due to tuberculosis (n=3) and posterior urethral valve (PUV) (n=1). The mean interval between EC and renal transplantation was 9.2 (range: 6.5–17) months.

Eight (6 male and 2 female) patients with abnormal bladder underwent UC (group B) using one (n=6) or two (n=2) dilated ureters. The mean age of these patients was 11.5 (range: 6–28) years. The etiologies of bladder dysfunction in this group included neurogenic bladder (n=7) and PUV (n=1). The mean interval between UC and renal transplantation was 7 (range: 6–14.5) months.

We performed EC by detubularized ileum, colon or sigmoid segment and in children, especially in boys, to facilitate the use of clean intermittent catheterization (CIC) (n=16), we transferred appendix on the augmentation bladder as conduit (Mitrofanoff procedure). 

For UC, we used one or two detubularized ureters after extraperitoneal nephrectomy. In anuric cases in the period between EC or UC and renal transplantation, cycling washout of the augmented bladder was performed via urethra (in adults by distilled water and in children by Mitrofanoff stoma). 

In this study, we also selected 30 kidney recipients who had had normal lower urinary tract (group C) as control group. This group included 22 males and eight females with a mean age of 15.6 years. Immunosuppressive drugs included cyclosporine, prednisolone and mycophenolate mofetile or azathioprine. In this study, we assessed the number of episodes of fever contributed to urinary tract infection (UTI) or hospitalization. In addition, graft loss and mortality rate in each group were compared with another.

Statistical analysis was performed by SPSS^®^. All data are presented as mean±SD. Qualitative variables were compared by χ^2^ or Fisher’s exact test, when appropriate. We used one-way analysis of variance (ANOVA) to compare means of continuous variables among the three studied groups. Kaplan-Meier survival analysis and log-rank test were used for assessing patients and graft survival. Cox proportional hazards model was used to assess the effect of selected factors including age, gender and the method used for bladder augmentation, on patient and graft survival.

## RESULTS

The mean follow-up time for groups A, B and C was 82, 63 and 72 months, respectively (overall, 73.1) (not significantly different). The demographic and clinical data of the three studied groups are shown in Table 1. Although the mean age of group A patients was significantly (p=0.009) higher than the group B, there was no significant difference between either of groups A and B with that of the comparison group. Episode of urosepsis (pyelonephritis) which resulted in hospitalization of recipients occurred 23 times in group A, six in group B and two times in C patients. There were significant differences in the rate between groups A and B (p=0.025) and groups A and C (p<0.001) but not between groups B and C (p=0.31). One-way ANOVA revealed no significant differences among the three studied groups in terms of follow-up period, and mean serum creatinine level.

**Table 1:** The demographic, clinical and laboratory data of study groups

Variable	Enterocystoplasty	Urterocystoplasty	Normal bladder
N	16	8	30
Gender			
Male	11	6	22
Female	5	2	8
Mean±SD age (yrs)	17.93±7.35*	11.50±4.34	15.63±3.35
Etiology of bladder dysfunction			
Neurogenic Bladder	12	7	–
Contracted Bladder due to tuberculosis	3	0	–
Posterir urethral valve	1	1	–
Mean±SD follow-up (months)	82±45	63±39	72±41
Episodes of febrile UTI	29^†^	6	2
Mean±SD serum creatinine (mg/dL)	1.72±0.31	1.37±0.13	1.33±0.59
Normal graft function	11	7	24

* Compared with urterocystoplasty (p=0.012).

† Compared with urterocystoplasty (p<0.03) and with normal bladder (p<0.01).

**Figure 1:**
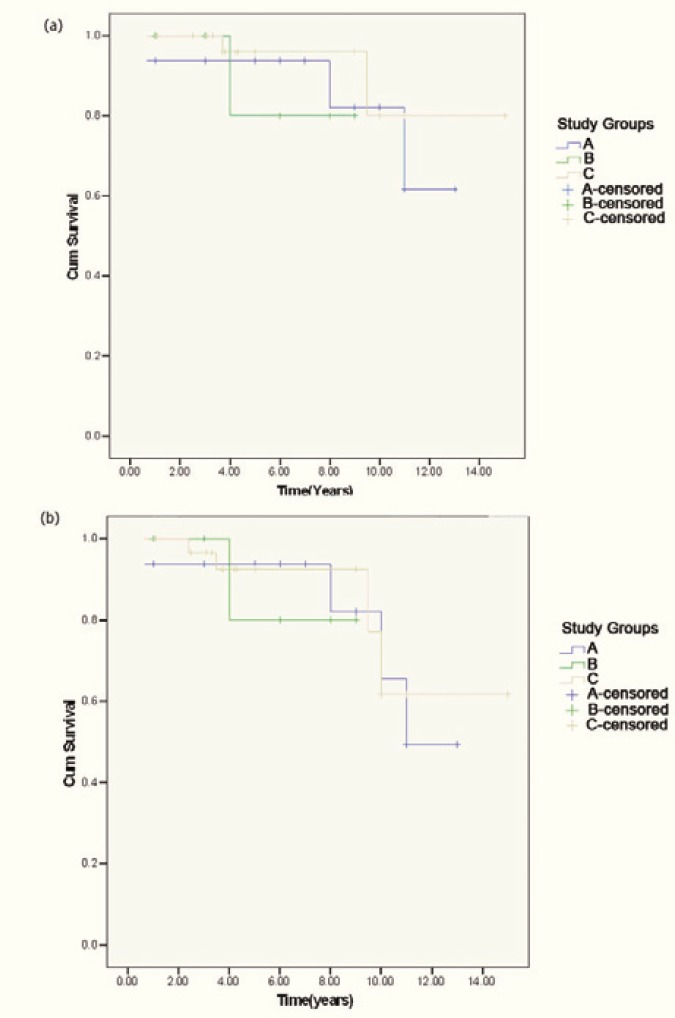
Kaplan-Meier estimates for renal transplant recipients with enterocystoplasty (group A, n=16), uretrocystoplasty (group B; n=8), and normal bladder (group C; n=30). Log-rank test, a) Graft survival, p=0.875; and b) Patient survival, p=0.645

In group A, the cause of graft loss in three patients was chronic rejection and in two was pyelonephritis. In group B, graft loss occurred due to chronic rejection in two patients and pyelonephritis in one patient. In group C, six patients lost their graft due to chronic rejection. Table 2 shows the overall 1-, 5- and 10-year graft and patient survival among the three studied groups. Figure 1 shows the Kaplan-Meier cumulative graft and patient survival curves among the three studied groups. There were no significant differences among the three studied groups. Cox proportional hazards model showed no significant effect of gender, age and type of bladder augmentation on the graft survival (Table 3). Two patients in group A died of urosepsis and one of group B patients died of gastrointestinal problem; graft function in these cases was normal. In group C, one patient died of liver disease and one of cardiovascular problem. 

**Table 2:** Graft and patient survival among the three studied groups

Variables	Enterocystoplasty	Urterocystoplasty	Normal bladder
Graft Survival			
1-year	94%	100%	100%
5-year	82%	80%	92%
10-year	66%	80%	61%
Patient Survival			
1-year	94%	100	100%
5-year	94%	80%	96%
10-year	82%	80%	84%

**Table 3:** Cox proportional hazards model multivariate analysis for factors predictive of graft and patient survival

	Graft Survival		Patient Survival	
Variables	Hazard Ratio	P Value	Hazard Ratio	P Value
Gender	0.402	0.206	0.288	0.176
Age	1.091	0.147	1.145	0.08
Enterocystoplasty	1.036	0.963	3.131	0.860
Urterocystoplasty	3.131	0.357	7.597	0.155

## DISCUSSION

Our study shows that augmentation cystoplasty with segment of intestine or dilated ureter is a safe and effective procedure for reconstructing lower urinary tract before renal transplantation. In recipients with EC, the frequency of febrile UTI and urosepsis is high so that it may become dangerous. The study also shows that graft and patient survival rate in kidney recipients with pre-transplant EC or AC is almost similar to that of kidney recipients who have normal lower urinary tract.

When renal failure results from underlying urologic anomalies (*e.g.*, PUV, prune belly syndrome, neurogenic bladder, *etc*) it can be assumed that the abnormal bladder contributed to the destruction of the native kidneys, might adversely influence the outcome of the transplanted graft too [4]. Moreover, many reports have shown that bladder dysfunction can negatively affect graft function if left untreated [5].

Augmentation cystoplasty with segment of intestine or dilated ureter is an optional procedure for reconstruction of low capacity or poorly compliant bladder [4]. In 1982, Marshall, *et al*, reported the first augmentation cystoplasty after kidney transplantation in a man [6]; in 1984, Steplanson, *et al*, reported the first pediatric kidney transplant drained to an augmented bladder [7]. 

Our first bladder EC with segment of ileum was performed in 1993 in a 12-year-old patient with neurogenic bladder. Six months after this operation, renal transplantation was performed. This patient was admitted two times due to urosepsis within the first three months of renal transplantation. After five patients of EC we performed the first UC in 1997, in a 7-year-old boy.

EC cases (group A) had more complication rates after renal transplantation in comparison with UC cases (group B), especially in terms of symptomatic UTI and urosepsis and the frequency of hospitalizations. Two recipients from EC group died of urosepsis nine months and five years after renal transplantation. Therefore, we emphasized on UC in those who had abnormal bladder with dilated ureter. In 1997, Alfrey and co-investigators reported their results of augmentation cystoplasty in 10 children with renal failure, but the rate of catastrophic complications were so high that they advised ileal conduit instead of augmentation [8]. Nevertheless, the current and other studies suggest that transplantation can be safely performed in patients with reconstructed bladder with acceptable graft survival and function [9-11]. Our study shows that in long-term, there is no significant differences in graft and patient survival rate between the studied augmentation (EC and UC) and the comparison groups. Nahas, *et al*, reviewed 25 renal transplant recipients with bladder dysfunction who underwent augmentation cystoplasty and reported that 20 (80%) kidneys survived after a mean follow-up of 53.2 months [2] . One-, 2-, and 5-year graft survival rate was 96%, 92% and 78%, respectively. Complications included symptomatic UTI, ureteral stenosis, and lymphocele. It is concluded that augmentation cystoplasty is safe and effective for restoring lower urinary tract function in transplant recipients who have a small noncompliant bladder [2]. Similarly, Zaragosa, *et al*, reviewed 11 renal transplant recipients who underwent augmentation cystoplasty and reported that nine grafts were functioning after a mean follow-up of 30.1 months [12]. Aki, *et al*, also reported that renal transplantation can be performed safely after augmentation cystoplasty [13].

There are some complications after augmentation cystoplasty and renal transplantation including symptomatic UTI, metabolic acidosis, ureteral stenosis and urinary calculi. In our patients, symptomatic UTI and urosepsis were more prevalent in EC than UC and normal bladder groups; it may be attributed to the fact that the bladder which is augmented with segment of intestine is commonly colonized with enteric flora. As a result, in most of these cases, urine cultures were positive but there was no symptomatic UTI or symptomps of pyelonephritis. Most studies recommend not treating asymptomatic UTI three months after renal transplantation. An exception is infection with *Proteus mirabilis* which leads in formation of struvite stones [14]. Nfild, *et al*, reported that symptomatic UTI was more common in the first three months after renal transplantation (63%) and during the period of fever. They also reported that systemic symptoms occurred in 39% of recipients with normal bladder and 59% of recipients with abnormal bladder [4]. We know that UTI in abnormal bladder directly affects graft. Therefore, patients with EC who undergoes kidney transplantation are regarded as especial cases if symptomatic UTI and deterioration of graft function develop, so repeated urinary cultures during the first three months and prophylactic antibiotic administration is recommended. However, three months after renal transplantation, asymptomatic UTI does not need any treatment [4].

The timing of the augmentation related to renal transplantation is a controversial issue. We prefer to perform augmentation before renal transplantation to prevent graft damage by the high intravesical pressure of the low compliant bladder; in this study all augmentations for the two studied groups A, and B were performed before renal transplantation. One author suggests performing augmentation for anuric patients 3–6 months after renal transplantation to avoid complications related to dry cystoplasty [15]. In anuric cases, particularly in those who are expecting cadaveric graft, washing cyclically of the augmented bladder by cyclically CIC is necessary. In the current study, CIC was performed for 16 recipients. Although that results in bacteriuria in virtually all the 16 cases, the safety of CIC and renal transplantation has withstood the test of time [16,17].

## CONCLUSION

Our study demonstrates that kidney transplantation into augmentation bladder is safe and effective. UTI is the most common complication after renal transplantation in recipients with augmented bladder. Urosepsis is more frequent in recipients with EC than UC and can sometimes be dangerous. In long-term, bladder augmentation does not adversely affect survival of patient and graft.
